# Population Genetic Diversity and Viability of the North China Leopard ( *Panthera pardus japonensis* ) in the Eastern Loess Plateau, China

**DOI:** 10.1002/ece3.71150

**Published:** 2025-03-19

**Authors:** Xinyue Wen, Hailong Dou

**Affiliations:** ^1^ College of Life Science Qufu Normal University Qufu China; ^2^ School of Life Sciences Fudan University Shanghai China

**Keywords:** fecal DNA, genetic diversity, *Panthera pardus japonensis*, population viability analysis

## Abstract

The North China leopard population in the eastern Loess Plateau, China, is highly fragmented and faces an increased risk of extinction due to habitat loss and environmental change. Therefore, timely research on leopard conservation in the Loess Plateau region can provide scientific guidance for its management and population recovery. To investigate the impacts of habitat fragmentation on genetic diversity, we analyzed 129 fecal samples from the eastern foot of the Loess Plateau, China. Using eight pairs of microsatellite loci and mitochondrial *ND‐5* gene sequences, we genetically identified 41 individual leopards. Analysis of microsatellite data showed an intermediate level of genetic diversity (*PIC* = 0.60). Based on the *ND‐5* gene sequence, five haplotypes were identified, with a moderate level of genetic diversity (*Hd* = 0.23, *Pi* = 0.00048). Inbreeding analyses documented significant population inbreeding pressure. Using VORTEX, we also explored the future development of the population by analyzing the population viability. The results indicated no extinction risk for the leopard population in the next 100 years under current genetic conditions. However, a loss of 22% genetic diversity is expected. These results emphasize the need to strengthen leopard monitoring, mitigate inbreeding, and restore habitat connectivity to preserve genetic diversity.

## Graphical abstract

A survey on the genetic status of the North China leopard population in fragile habitats, the results of which indicate that the North China leopard population in the eastern Loess Plateau is undergoing a process of genetic decline.

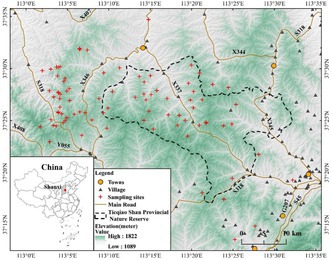

## 1 Introduction

Habitat fragmentation can impede gene flow between populations, leading to reduced genetic diversity and increased inbreeding pressure, which can ultimately result in species extinction (Haag et al. 2010; Khalatbari et al. 2023). Anthropogenic disturbance is currently the main cause of wildlife habitat fragmentation on a global scale, and habitat fragmentation has a particularly significant impact on large carnivores with wide ranges and sparse populations (Ripple et al. 2014; Schlaepfer et al. 2018). Inbreeding reduces the genetic diversity of small populations, which in turn reduces the ability of populations to adapt to their environment and increases the risk of extinction (Camus et al. 2023). Therefore, investigating genetic diversity and population viability is crucial for developing or improving management and conservation measures for endangered species.

Population viability analysis (PVA) can simulate population trends under different environmental conditions using a variety of models (Morrison et al. 2016). It predicts future population trends by assessing the relationship between population size and factors like climate and disease and estimates the minimum viable populations of endangered species needed to avoid extinction. The PVA provides a scientific basis for reducing extinction risk by predicting both the probability and timing of extinction while identifying key factors contributing to population extinction. This information is crucial for assessing the status of endangered species and informing management plans (Lacy 2019; Zeigler et al. 2013). Despite ongoing controversy over the model assumptions, the reliability of the data, and the interpretation of the results, PVA remains an important tool for wildlife conservation and prediction of population trends (Chaudhary and Oli 2020). However, combining PVA with other biological methods can improve accuracy and help guide conservation and management efforts.

The North China leopard ( *Panthera pardus japonensis* ), endemic to China, is one of nine leopard subspecies, with its core distribution area in the Loess Plateau (Jacobson et al. 2016; Laguardia et al. 2017). As the only top predator in this region, it plays an important role in maintaining the functional stability of the Plateau ecosystem (Fu et al. 2023; Yang et al. 2021). However, habitat destruction and increased anthropogenic disturbance have significantly reduced its range, putting the leopard population at high risk of extinction. To protect this flagship species, the IUCN listed it in the Red List of Threatened Species, and the Chinese government designates it as a national‐level protected species (Song et al. 2014).

Broadly, the distribution of the North China leopard in the Loess Plateau can be divided into two regions. The first is the Shanxi–Gansu–Ning–Qinling area, west of the Yellow River in the west‐central Loess Plateau, with the largest known population in Ziwuling Nature Reserve, Shaanxi Province. The second is the North China region, dominated by the Taihangshan in the eastern Loess Plateau (Yang et al. 2021). While most available ecological studies focus on the central and western regions (Yin et al. 2023; Zeng et al. 2022), studies on the eastern population are lacking. The eastern population faces more severe threats from fragmentation and anthropogenic disturbances (Zhao et al. 2020), highlighting the urgent need for studies to assess its status and guide conservation efforts.

In this study, we used a noninvasive sampling method to systematically investigate the genetic diversity and population viability of the North China leopard population in the eastern Loess Plateau and to answer the following questions: (1) What is the population size and genetic diversity of the leopards in this region? (2) Is the population inbred, and has it historically experienced population bottlenecks? (3) What is the likelihood of future extinction, and what key factors affect population development?

## 2 Materials and Methods

### 2.1 Study Area

**FIGURE 1:**
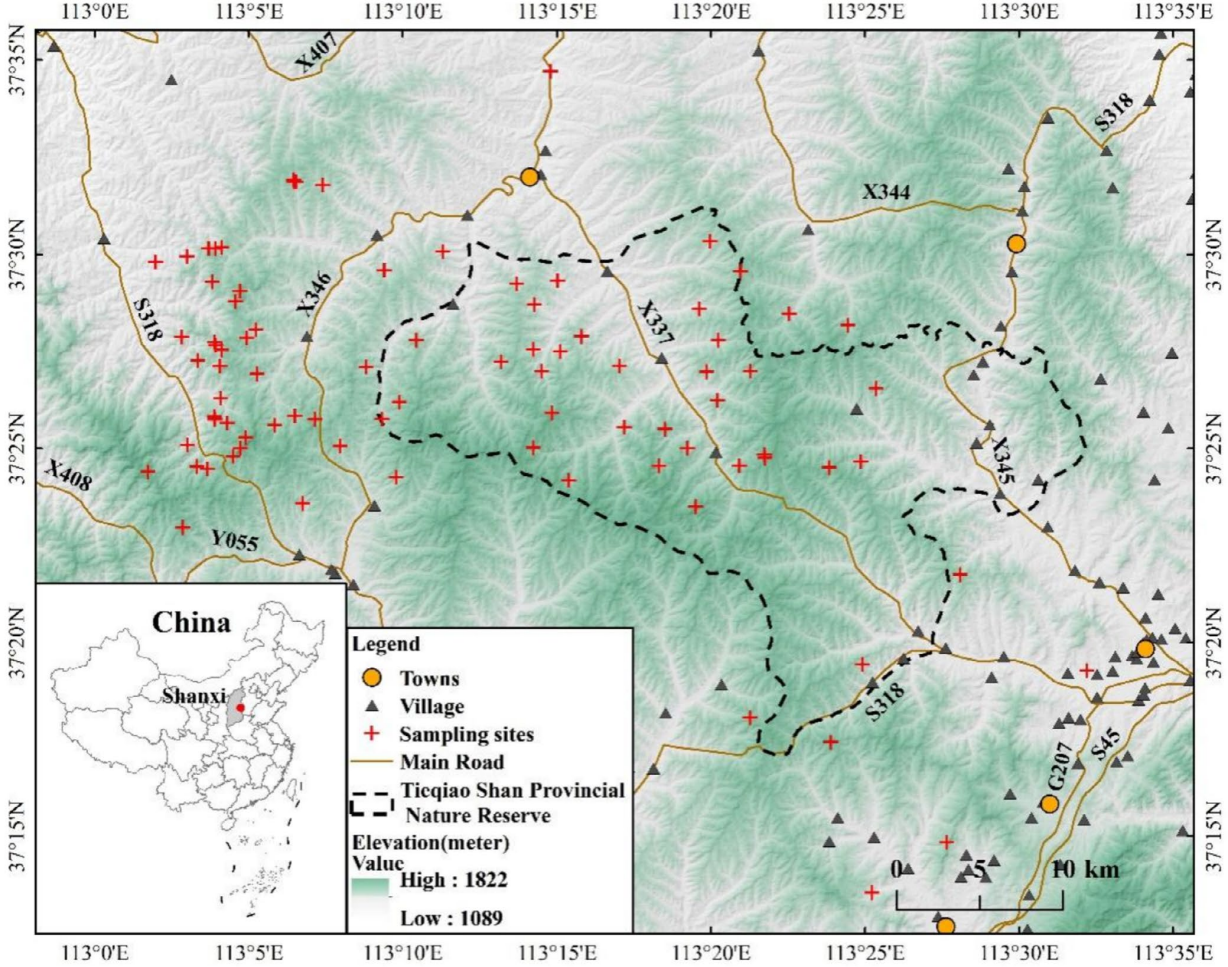
Location of leopard fecal samples collected from the Tieqiao Mountain Provincial Nature Reserve and surrounding areas in the eastern Loess Plateau of China.

The study area is located in the eastern part of the Loess Plateau, encompassing the Tieqiaoshan Nature Reserve and its surrounding areas in Shanxi Province, China (113°00′‐113°30′ E, 37°20′‐37°32′ N), with a total area of 1808 km^2^ (Figure 1). The main conservation targets of the reserve include the North China leopard and the natural forest ecosystem in which it is located. The area has mountainous topography with 40.2% forest cover and a temperate continental climate, which experiences an average annual temperature of 6.3°C and receives an annual precipitation of 593 mm (Song et al. 2014; Vitekere et al. 2020). The vegetation in the region is dominated by deciduous broad‐leaved forests, coniferous forests, and deciduous mixed forests. The area supports diverse wildlife, including 24 species of mammals, six species of reptiles, three species of amphibians, and 116 species of birds. The North China leopard mainly preys on wild boar ( *Sus scrofa* ), siberian musk deer ( *Moschus moschiferus* ), and roe deer ( *Capreolus pygargus* ) (Vitekere et al. 2020; Zheng et al. 2009; Zhu et al. 2021). However, the region faces significant habitat degradation and fragmentation due to human activities, including farmland, human settlements, and physical infrastructure construction (e.g., roads) (Consolee et al. 2020) (Figure 1).

### 2.2 Sample Collection

Between November 2020 and June 2023, we collected 158 fecal samples from areas with frequent leopard activity. To prevent cross‐contamination, sampling tools were replaced after each collection. The sources and freshness of the feces were determined using morphological characteristics and nearby signs of leopard activity, such as footprints and recumbent and scavenging marks. Only fresh and intact fecal samples were selected, collected at a minimum distance of 1 km, and stored in 50 mL centrifuge tubes with anhydrous ethanol. Each fecal sample was labeled with geographic coordinates, sampling date, and sample number, then refrigerated at −20°C for further analysis.

### 2.3 Extraction of Fecal DNA and Species Identification

Fecal DNA was extracted using the QIAamp Stool mini Kit (QIAGEN, Hilden, Germany). Species identification was performed by amplifying and sequencing fecal DNA using the vertebrate universal primer 16S‐F/R (targeting the mitochondrial 16S rRNA gene) and the leopard‐specific primer Ppo‐CbF/R (targeting the cytochrome b gene) (Sugimoto et al. 2006; Xiong et al. 2016). The 20 μL PCR system consisted of 10 μL dNTP mixture, 0.1 μg/μL BSA, 0.6 μL each of forward and reverse primers, 2 μL DNA template, and ddH_2_O to reach the final volume. The PCR protocol included predenaturation at 95°C for 5 min, followed by 40 cycles of denaturation at 95°C for 30 s, annealing at 50°C–56°C for 30 s, and extension at 72°C for 30 s. The reactions were performed under sterile conditions to prevent cross‐contamination. PCR products were purified and sent to Shanghai Biotech for sequencing. Sequences were analyzed using BLAST described on the website of the National Center for Biological Information (https://www.ncbi.nlm.nih.gov/) for species identification.

### 2.4 Microsatellite and Mitochondrial Gene Amplification

We selected eight pairs of microsatellite loci with high amplification rates and polymorphisms for individual identification and genetic analysis of North China leopards (Mondol et al. 2009; Spencer et al. 2016; Waits et al. 2007) (Table S1). Sex determination was performed by amplifying the amelogenin region with primers designed by Pilgrim et al. (2005). The PCR conditions were the same as those used for species identification, with both negative and positive controls. The positive control sample came from the muscle tissue of a male leopard killed in a road traffic accident. Due to low DNA content in fecal samples, PCR amplification was prone to typing errors. Therefore, we employed a multi‐tube amplification method to improve accuracy in interpretation (Taberlet et al. 1996). Heterozygotes were identified by two identical typing results, and pure heterozygotes by three identical typing results. PCR products were sent to Shanghai Biotech for capillary electrophoresis sequencing using ABI 3730 XL automated sequencer (Applied Biosystems Inc). Sequencing results were read and corrected using GeneMapper 4.1, followed by manual proofreading.

Additionally, we amplified and sequenced 611 bp fragments of the mitochondrial *ND‐5* gene using amplification primers and methods described in Uphyrkina et al. (2001). The PCR‐amplified products were sent to Shanghai Biotechnology for sequencing. Mitochondrial DNA sequences were analyzed with Codoncode Aligner 3.6.1 software (http://www.codoncode.com/aligner/) and further edited using Clustal X (Larkin et al. 2007), with additional manual proofreading.

### 2.5 Analysis of Population Genetic Diversity

We used CERVUS 3.0 (Kalinowski et al. 2007) to analyze and compare sample genotypes, determining the number of individuals based on genotypic matches at a minimum of six loci (Dou et al. 2016). Probability of Identity (PID) values were calculated using GIMLET 1.3.3 (Valière 2002) for joint identification of microsatellite loci. We used the software MICRO‐CHECK (Van Oosterhout et al. 2004) and GIMLET 1.3.3 to detect the presence of null alleles and allele loss at each microsatellite locus. Genetic diversity metrics, including the number of alleles (*Na*), observed heterozygosity (*Ho*), expected heterozygosity (*He*), and polymorphic information content (*PIC*), of microsatellite loci were calculated using CERVUS 3.0. Genepop 3.4 (Rousset 2008) was used to determine genetic diversity and detect Hardy–Weinberg equilibrium and linkage disequilibrium (*LD*) (Rozas et al. 2003). Additionally, haplotypes of the mitochondrial *ND‐5* gene were analyzed, and haplotype diversity (*Hd*) and nucleotide diversity (*Pi*) were calculated using DNASP (version 6.0) (Petit et al. 1998). In order to visualize the phylogenetic relationships among the various populations in the Loess Plateau, we downloaded the *ND‐5* gene haplotypes (Genbank Accession Number: ON822089‐92) (Yin et al. 2023) of the North China leopard from the central part (Ziwuling Nature Reserve, *ZNR*) and the western part (Liupanshan Nature Reserve, *LNR*) and constructed a haplotype network using the median‐joining method in NETWORK version 5.0 (Bandelt et al. 1999).

We also used the interlocking disequilibrium (*LD*) method (Waples and Do 2010), suitable for calculating the effective population size of small populations, via NeESTIMATOR 2.0 to calculate the effective population size (*Ne*) (Gilbert and Whitlock 2015). Confidence intervals (95%) were estimated using the scissor (Jackknife) method to mitigate bias from low gene frequencies (Waples and Do 2008); data with alleles ≤ 2% were discarded.

### 2.6 Detection of Inbreeding and Population Bottleneck Effects

To investigate inbreeding, we analyzed the microsatellite data using Lynch & Ritland's method, that is, Coancestry 1.0.1.7 software (http://www.zsl.org/science/research/software/) and calculated the inbreeding coefficients of individual leopards (Lynch and Ritland 1999). An inbreeding coefficient greater than 0 indicates proximity stress, while a coefficient less than 0 indicates no proximity stress.

We used Bottleneck 1.2 software (Piry et al. 1999) to test for a recent population bottleneck in the North China leopard population. The software compares expected heterozygosity with allele numbers, assuming mutation‐drift equilibrium to determine whether a population has experienced a bottleneck effect (Du et al. 2016; Luikart et al. 1998b). Three models were selected: the infinite allele model (*IAM*), the stepwise mutation model (*SMM*), and the two‐step mutation model (*TPM*). A Wilcoxon rank test with 1000 simulations was used to test for significant excess heterozygosity. Also, allele frequency distribution plots (mode‐shift metrics) were used to test bottlenecks (Luikart et al. 1998a), where the L‐shaped distribution indicated no population bottleneck.

### 2.7 Population Viability Analyzes

We used VORTEX 10 (Lacy 1993), an individual‐based stochastic modeling software, to simulate the population viability of the North China leopard population. VORTEX models population dynamics under specific environmental conditions and evaluates the effects of different stochastic events, helping to assess extinction risk. Due to the scarcity of data related to the North China leopard, the biological parameters entered in the software are based on data from other leopard subspecies studies (Balme et al. 2013; Daly et al. 2005; De Vos et al. 2001; O'Grady et al. 2006). Females typically give birth to 2–3 cubs per litter, with an assumed equal sex ratio. The simulation did not account for the occurrence of catastrophic events due to the strong survivability of the North China leopard, the absence of natural predators, sufficient prey, and minimal population fluctuations. Using prey densities from infrared camera data (unpublished) in the study area, we estimated the carrying capacity of the area to support a maximum of 70 leopards. The simulation ran for 100 years with 1000 iterations, defining extinction as a population with only one remaining sex (refer to the parameters in Table 1).

**TABLE 1:** Initial simulation parameters for the viability analysis of the North China leopard population in Shanxi Province, China.

Parameters	Numerical value	
	Female	Male
Number of iterations	1000	
Number of years	100	
Extinction definition	Only one sex remains	
Lethal equivalents	6.29	
Dispersal	No	
Reproductive system	Polygynous	
Age of first offspring	3	3
Maximum age of reproduction	14	14
Maximum lifespan	16	16
Maximum number of progeny per brood	3	
%males in breeding pool		70
Sex ratio at birth – in % males	50	
%adult females breeding	50	
Morality rates %	40 (0–1 year old)	40 (0–1 year old)
	10 (2–3 year old)	14 (2–3 year old)
	5 (> 6 years old)	7 (> 6 years old)
Catastrophy	No	
Harvest	No	
Initial population size	40	
Carrying capacity K	70	

We conducted sensitivity analyses on uncertain modeling factors, simulating population dynamics by varying key parameters to identify those with the greatest impact on population development. Lethal equivalents were set to 3.14, 6.29, and 12.58 (O'Grady et al. 2006), while the rest of the variables were adjusted by ±40% of their set values (He et al. 2020). Sensitivity indices were calculated, with higher values indicating greater parameter impact on the simulation. The sensitivity index was calculated using the following formula:

$$\mathrm{Sr}=\left(\Delta r/r\right)/\left(\Delta P/P\right)$$

where Δ*r*/*r* represents the change in the mean population growth rate, and Δ*P*/*P* represents the change in the parameter (Pulliam et al. 1992).

## 3 Results

### 3.1 Individual Identification and Population Genetic Diversity

**FIGURE 2:**
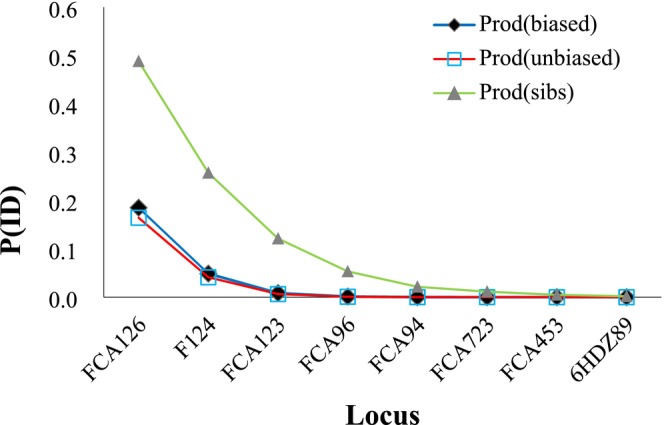
Probability of joint identification (*PID*) of microsatellites.

Of158 feces samples, 129 were identified as leopards, of which 48 were successfully genotyped, resulting in a genotyping success rate of 37.21%. In total, 41 individuals were identified, comprising 25 males and 16 females, with a female‐to‐male ratio of 0.64. The *PID* results showed a high joint recognition rate across the eight microsatellite loci, allowing for effective individual identification (Figure 2). The MICRO‐CHECKER analyses revealed null alleles at loci FCA126, FCA123, FCA096, FCA094, FCA453, and 6HDZ89. The average number of alleles in the leopard population was 6.75, with the highest number at locus 6HDZ89 (9 alleles) and the lowest at locus FCA126 (5 alleles) (Table 2). The observed heterozygosity across the eight loci ranged from 0.35 to 0.67, with an average expected heterozygosity of 0.65. The mean polymorphic information content (*PIC*) was 0.59, with individual loci having PIC values around or above 0.50, demonstrating high polymorphism (Luo et al. 2019). None of the loci deviated from Hardy–Weinberg equilibrium or showed signs of linkage disequilibrium.

**TABLE 2:** Genetic information of microsatellite loci in the North China leopard population.

Locus	*Na*	*Ho*	*He*	*PIC*
FCA126	5	0.350	0.561	0.521
F124	7	0.587	0.573	0.484
FCA123	6	0.479	0.644	0.588
FCA096	7	0.422	0.683	0.623
FCA094	7	0.667	0.763	0.717
FCA723	6	0.429	0.542	0.491
FCA453	7	0.514	0.740	0.682
6HDZ89	9	0.476	0.692	0.653
Average	6.750	0.4905	0.6479	0.5949

Abbreviations: He, expected heterozygosity; Ho, observed heterozygosity; Na, number of alleles; PIC, polymorphic information content.

The mitochondrial *ND‐5* gene fragment was successfully amplified to obtain 33 leopard individual sequences with a length of 496 bp. The nucleotide composition showed a 60.90% A + T content, indicating a base bias. Mitochondrial genetic diversity showed that haplotype diversity (*Hd*) was 0.23 and nucleotide diversity (*Pi*) was 0.00048.

**FIGURE 3:**
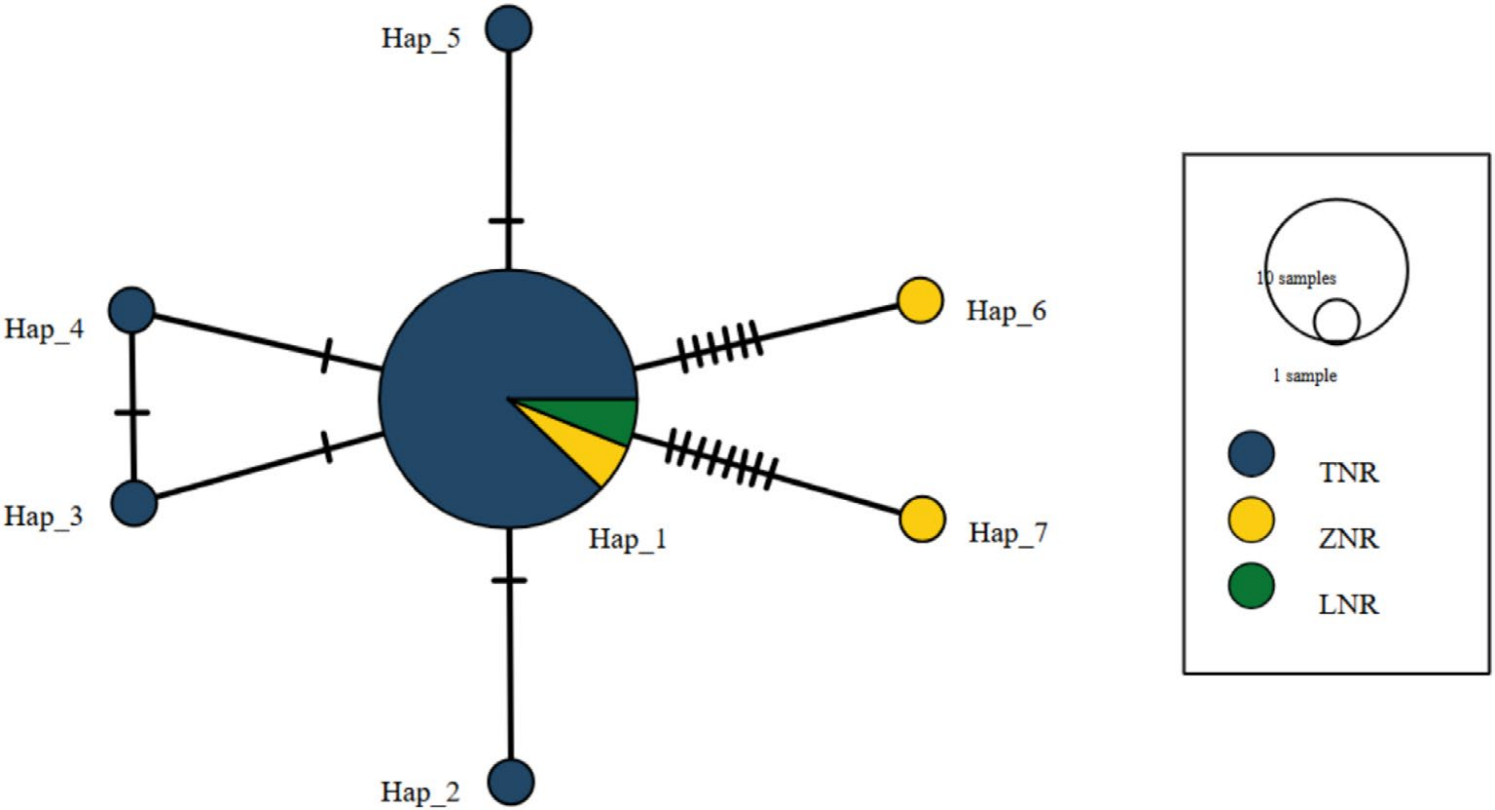
Median‐joining haplotype network of the North China leopard populations in the Loess Plateau, China. Each unique haplotype is represented by a circle. Circle size indicates the number of individuals and each transverse dash on a line indicates a substitution between haplotypes Blue color indicates the haplotypes of the leopard population in the eastern part of the Loess Plateau, yellow represents the central part, while green represents the western part.

Sequence similarity analysis revealed 15 polymorphic sites, identifying five haplotypes. Among them, *Hap1* was the most common, occurring in 87.88% of individuals, while the other four haplotypes were each found in only one individual. The haplotype network diagram showed that the eastern population (*TNR*), where the study was conducted, had the highest number of haplotypes, followed by the central population (*ZNR*), and the western population (*LNR*) had the lowest number of haplotypes (Figure 3). Haplotype 1 was found in all populations, and the other six haplotypes were population‐exclusive haplotypes.

The NeEstimator results showed that the effective population size of the North China leopard population was 12.20 (95% CIs: 8.90–16.70). With 41 identified leopard individuals, the ratio of effective to actual population size (*Ne*/*Nc*) was 0.30.

### 3.2 Determining Effects of Inbreeding and Bottleneck

The Coancestry analysis showed that 32 out of 41 leopards had an inbreeding coefficient greater than 0, while only nine had coefficients below 0 (Table S2). The Lynch & Ritland method yielded an average inbreeding coefficient (*LR‐estimator*) of 0.21, indicating a moderate level of inbreeding in the population.

The BOTTLENECK results showed no evidence of a bottleneck event in the leopard population under the *IAM* and *TPM* models (*p* > 0.05). However, under the *SMM* model, the population may have experienced a recent bottleneck (*p* < 0.05). The L‐shaped allele frequency distribution in the mode shift further indicated no recent bottleneck event (Table 3).

**TABLE 3:** Bottleneck test information for the North China leopard population.

Model	*IAM*	*SMM*	*TPM*	Mode‐shift
	0.074	0.004	0.074	L‐shape

### 3.3 Population Viability Analyzes

**FIGURE 4:**
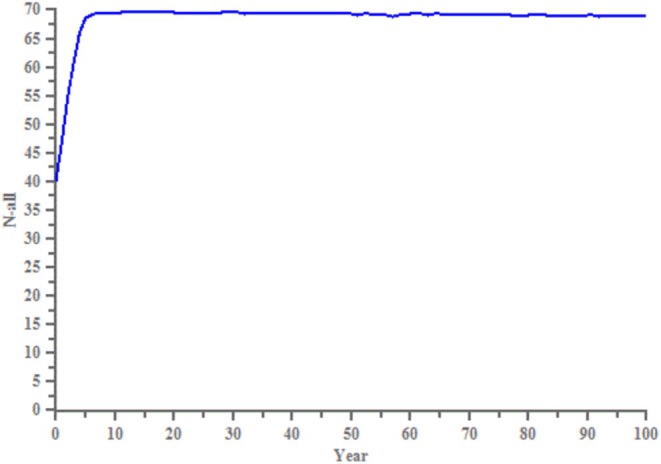
Trends in population size of the North China leopard in the eastern foothills of the Loess Plateau over a 100‐year period. Horizontal coordinates represent time; vertical coordinates represent the population size.

The VORTEX simulation showed no extinction event for the leopard population in the region within 100 years. By the end of the simulation, the population grew to 69 (Figure 4), nearing the initial carrying capacity, with an average growth rate of 0.11 (95% CIs: 0.10–0.11). The genetic diversity, starting at 1 (default initial value), declined to 0.78.

The sensitivity analysis showed that the average growth rates were 0.12, 0.11, and 0.08 when lethal equivalents were set to 3.14, 6.29, and 12.58, respectively, indicating a decreasing trend. After simulation, the population sizes were 69, 69, and 66, respectively, with a sensitivity index of 0.57. The breeding rate of adult females and the sex ratio at birth had the highest sensitivity indices (1.45 and 1.43), making them the most influential factors in the development of the North China leopard population (Table 4). There was a 1% probability of extinction when the breeding rate of adult females decreased by 40%, and a 5% probability when the sex ratio at birth increased by 40%. The sensitivity index for the juvenile female mortality rate was 0.93, indicating a significant impact, while juvenile male and adult male mortality rates had low sensitivity indices (0.02), indicating minimal influence on the North China leopard population development.

**TABLE 4:** Sensitivity results of different parameters of the North China leopard population.

Parameters	N		*r*		Sensitivity index (*Sr*)
	−40%	+40%	−40%	+40%	
Lethal equivalents	69(3.14)	66(12.58)	0.123(3.14)	0.075(12.58)	0.5660
Carrying capacity K	40	79	0.090	0.113	0.2712
Sex ratio at birth	69	53	0.149	0.028	1.4269
Breeding rate of adult females	56	70	0.032	0.155	1.4505
Mortality rate of juvenile females	69	66	0.140	0.061	0.9316
Mortality rate of juvenile males	69	69	0.105	0.105	0.0236
Mortality rate of adult females	69	68	0.117	0.094	0.2712
Mortality rate of adult males	69	69	0.107	0.105	0.0236

*r* indicates mean stochastic grow rate. −40% and + 40% indicates minus and plus 40% of base value. Figures in parentheses indicate different lethal values.

## 4 Discussion

### 4.1 Individual Identification

The genotyping success rate of the North China leopard is lower than that of other leopard subspecies and large carnivores (Dou et al. 2016; Sugimoto et al. 2014a; Wang et al. 2016). The success varied significantly from the time of fecal deposition to collection and the surrounding environment, but not from the time of fecal collection to DNA extraction or the month of collection (Cho et al. 2022). Since North China leopards are mainly active in remote high mountainous areas with minimal human presence, most of the fecal samples collected were relatively old. The long deposition time causes fecal DNA degradation, which contributes to the reason for the low success rate of genotyping.

We used noninvasive genetic methods to identify a population of 41 leopards, consistent with a minimum population size of 40 observed by infrared cameras in 2022 (Li 2023). The infrared camera data also showed a high population turnover rate for the leopards in the region (Zhou et al. 2022). Our 2.5‐year fecal sample collection period may have contributed to an overestimate of the actual population. Of the 41 individuals, 60.98% were males, likely due to the larger territories of male leopards, which are, generally, 2–3 times that of females, increasing the chance of collecting male fecal samples.

### 4.2 Population Genetic Diversity

The genetic diversity of the North China leopard population is intermediate compared to other leopard subspecies, such as the Indian leopard (*P. p. fusca*) (*He* = 0.79), Indo‐China leopard (*P. p. delacouri*) (*He* = 0.73), Sri Lankan leopard (*P. p. kotiya*) (*He* = 0.49), and Amur leopard (*P. p. orientalis*) (*He* = 0.35) (Cho et al. 2022; Singh et al. 2022; Sugimoto et al. 2014b; Uphyrkina et al. 2001). In comparison with other North China leopard populations, leopards in this region have lower mean expected heterozygosity (*He* = 0.65) and mitochondrial gene diversity (*Hd* = 0.23) than those in the west‐central Loess Plateau (*He* = 0.72, *Hd* = 0.83) (Yin et al. 2023). This could be due to increased anthropogenic disturbances and habitat fragmentation, leading to reduced gene flow and lower genetic diversity. A mitochondrial haplotype network map identified five haplotypes in the mitochondrial *ND‐5* gene fragment, and most individuals are of the same haplotype, indicating low haplotype diversity, consistent with the findings for other leopard subspecies (Asad et al. 2019; Farhadinia et al. 2015). In contrast, large Asian carnivores like the brown bear and the Himalayan wolf show high haplotype diversity (Ashrafzadeh et al. 2016; Werhahn et al. 2018), likely due to later divergence and slower molecular evolution in felids (Kim et al. 2016).

Effective population size (*Ne*) represents the size of an ideal population that reflects the rate of genetic diversity loss due to genetic drift. It is typically much smaller than the actual population size (*Nc*), and the *Ne*/*Nc* ratio is a key indicator of the long‐term viability of a population (Gilbert and Whitlock 2015). Wildlife populations typically have mean *Ne*/*Nc* ratios of 0.10–0.11, with factors like population size, sex ratio, and variability in reproductive success leading to even lower ratios (Ferchaud et al. 2016; Palstra and Fraser 2012). The *Ne*/*Nc* ratio of 0.30 for the leopard population in the eastern Loess Plateau suggests some potential for evolutionary change. However, for small populations, *Ne* = 50 can minimize the negative effects of inbreeding (Palstra and Ruzzante 2008). With an *Ne* of just 12.20 in the study area, the leopard population faces a high risk of inbreeding and loss of genetic diversity.

### 4.3 Inbreeding and Bottleneck Effects

Small, isolated populations are more prone to inbreeding, which reduces fitness, increases the risk of fixing deleterious mutations, and adversely affects survival and recovery (Armbruster and Reed 2005; Camus et al. 2023). In addition, prolonged inbreeding can reduce genetic diversity (Ripple et al. 2014). In the present study, the observed heterozygosity of the leopard population was found to be lower than the expected heterozygosity, which may be due to inbreeding and nonrandom mating within the population. Further inbreeding assessment shows signs of inbreeding decline in the leopard population; however, a relatively high genetic diversity suggests that the population may be in the early stage of inbreeding decline (Yin et al. 2023).

The results from the Bottleneck analysis showed that only the *SMM* model detected a population bottleneck phenomenon, while the *IAM* and *TPM* models did not. Previous studies have shown that the *TPM* model may be more suitable for microsatellite data analysis than the *IAM* or *SMM* models (Kataria et al. 2010). In this study, the *TPM* model showed no bottleneck in the North China leopard population. This suggests that poaching has not caused a significant decline in the leopard population. The failure of the *IAM* and *TPM* models to detect population bottlenecks in leopards may also be due to deviations in allele size distribution, the limited number of microsatellite loci, or the presence of null alleles in the population (Luikart et al. 1998a; Peery et al. 2012).

### 4.4 Population Viability Analyzes

VORTEX simulation results showed that the leopard population in our study area has not experienced an extinction in the past 100 years and is close to its carrying capacity. However, to ensure the long‐term survival and development of the population, its genetic diversity should be maintained at over 90% of current levels (Gong et al. 2012). By the end of the 100‐year simulation, the heterozygosity of the leopard population declined by 22%. This decline, combined with inbreeding, suggests that the leopard population will continue to decline due to inbreeding in the future.

The results from the sensitivity analyses showed that the breeding rate of adult females, the sex ratio at birth, and juvenile female mortality had the highest sensitivity indices, consistent with the findings for other species, such as elephants and pandas (Gong et al. 2012; He et al. 2020). In polygynous species such as leopards, females are key to population growth, as they determine the number of offspring (Clutton‐Brock 2009). For small populations, the female reproductive rate impacts population growth more than the survival rate (Gong et al. 2012). In contrast, male mortality had little effect on population growth rates in our study, likely due to males' shorter lifespans, earlier maturity, and faster turnover.

In the lethal equivalent simulation, the population growth rate of the North China leopard population decreased as lethal equivalents increased, indicating sensitivity to population development. The default value of 6.29 lethal equivalents in the VORTEX model, based on O'Grady et al. (2006), may underestimate extinction risk in wild populations. Our findings suggest using higher lethal equivalents in population viability analyses when specific data are absent. Crnokrak and Roff (1999) noted that inbreeding decline in the wild can be up to seven times more severe than in captivity. As inbreeding declines, the number of lethal equivalents increases, requiring 12.58 lethal equivalents to simulate severe inbreeding.

Carrying capacity limits population growth by restricting the number of individuals, though the impact on growth rates is not always obvious. For species to thrive, sufficient habitat and food must be available. Zhu et al. (2021) reported a density of 4.23 North China leopards per 100 km^2^ in the protected area within our study region, with a population of about 18 leopards, the highest density in the region. Our study area covers both the protected area and its surroundings, which are five times larger than the protected area. The significant presence of leopards outside the reserve suggests that the environmental carrying capacity inside the reserve may have reached its upper limit. Future managers will need to strengthen monitoring of leopard populations outside protected areas and make timely adjustments to protected area boundaries and management strategies based on monitoring results.

### 4.5 Implication for Leopard Conservation

Protecting leopard habitat is the top priority for conserving the North China leopard. Since the 1980s, the establishment of protected areas and the implementation of conservation policies have led to a steady increase in leopard numbers in the Loess Plateau, with their range gradually expanding northward (He et al. 2020). The Tieqiao Shan Nature Reserve, located in the eastern part of the Loess Plateau, serves as a crucial corridor for the northward expansion of the North China leopard. However, the development of villages and towns, increased construction projects, and provincial highways have fragmented natural ecosystems, isolating habitats and hindering leopard migration and dispersal (Figure 1). Our study found that there is a certain degree of inbreeding in the leopard population in the area under the current environmental conditions. In order to reduce further inbreeding leading to a reduction or even loss of genetic diversity, local management should reduce the impact of human activities on leopard habitat in the future.

The conservation of the North China leopard also has been challenged by human‐animal conflict. The study area is densely populated, where most farmers rely on agriculture and animal husbandry. As the leopard's habitat expands, livestock predation has increased, leading to a significant loss in livestock and the local economy. Between 2015 and 2018, 173 livestock were predated within the protected area alone (Consolee et al. 2020), fueling negative attitudes toward leopard conservation. Leopards were found in a wide range of areas outside the reserve, suggesting that the impacts of leopards on the local population have extended outside the reserve and signaling that the previous management approach of locating leopards within the reserve is no longer appropriate for the current situation. The need to revise the reserve plan as soon as possible and to address the economic damage caused by leopards to the population is a key issue for managers.

## 5 Conclusion

In conclusion, this is the first study to evaluate the genetic diversity and population viability of the North China leopard in the eastern Loess Plateau. While the leopard population shows a moderate level of genetic diversity, it faces inbreeding pressure, putting it at high risk of future genetic decline. Therefore, to protect the leopard population, it is crucial to enhance conservation research and closely monitor population dynamics and genetic diversity. Constructing ecological corridors to improve connectivity within and beyond protected areas and, when necessary, introducing individuals from the western Loess Plateau could enhance genetic diversity in this region. Finally, this study provides valuable insights into the current genetic status of the leopard population in the eastern Loess Plateau, aiding more informed conservation and management efforts.

## Author Contributions

**Xinyue Wen:** conceptualization (equal), data curation (lead), investigation (equal), methodology (equal), resources (lead), software (lead), writing – original draft (lead). **Hailong Dou:** conceptualization (lead), data curation (lead), funding acquisition (lead), investigation (equal), methodology (equal), project administration (lead), writing – original draft (lead), writing – review and editing (lead).

## Conflicts of Interest

The authors declare no conflicts of interest.

## Supporting information


Table S1.

Table S2.

## Data Availability

The original data are contained within the article and appendix.
