# Supplementary material for: Population Genetic Diversity and Viability of the North China Leopard ( Panthera pardus japonensis ) in the Eastern Loess Plateau, China
Source: Ecol Evol. 2025 Mar 19;15(3):e71150. doi: 10.1002/ece3.71150 (PMC11922602; doi:10.1002/ece3.71150)
Supplement: Supplementary file 1 — Table S1. [file ECE3-15-e71150-s001.docx]

TABLE S1 Information on eight pairs of microsatellite loci in the leopard population genetic analysis

| Locus | Dye | Size range (bp) | Annealing temperature/℃ | Primer sequences |
| --- | --- | --- | --- | --- |
| FCA126 | TAMRA | 139-145 | 55.5 | F:GCCCCTGATACCCTGAATG  R: CTATCCTTGCTGGCTGAAGG |
| F124 | TAMRA | 208-249 | 52.3 | F: TGCTGGGTATGAAGCCTACT  R: ATTGCCTCAACTACCTAGGC |
| FCA123 | ROX | 137-149 | 56 | F: ACTGCGAGAGGACTTTCGAA  R: CTTCTGACAGGCTCCAGGTT |
| FCA096 | ROX | 184-224 | 55 | F:CACGCCAAACTCTATGCTGA  R: CAATGTGCCGTCCAAGAAC |
| FCA094 | ROX | 215-237 | 57.8 | F: TCAAGCCCCATTTTACCTTC  R: CACCTGAGCCAAAGGCTATC |
| FCA723 | ROX | ~100 | 56 | F: TGAAGGCTAAGGCACGATAGA  R:CGGAAAGATACAGGAAGGGTA |
| FCA453 | HEX | 186-198 | 54.5 | F:AATTCTGAGAACAAGCTGAGGG  R: ATCCTCTATGGCAGGACTTTG |
| 6HDZ89 | FAM | ~100 | 50.8 | F:GCATAAAACTCTAACACAGCATCT  R: TTCTGAAATAGGATTGGCAAA |
